# Effect of aqueous extract of *Arctium lappa *L. (burdock) roots on the sexual behavior of male rats

**DOI:** 10.1186/1472-6882-12-8

**Published:** 2012-02-01

**Authors:** Cao JianFeng, Zhang PengYing, Xu ChengWei, Huang TaoTao, Bai YunGui, Chen KaoShan

**Affiliations:** 1 School of Life Sciences, Shandong University, Jinan 250100, PR China; 2 National Glycoengineering Research Center, School of Life Sciences, Shandong University, 27 shandananlu, Jinan 250100, PR China; 3 Affiliated Hospital of Shandong University, Jinan 250012, PR China

## Abstract

**Background:**

*Arctium lappa *L. root has traditionally been recommended as an aphrodisiac agent. It is used to treat impotence and sterility in China, and Native Americans included the root in herbal preparations for women in labor. However, its use has not been scientifically validated. The present study therefore investigated the effects of aqueous extract of *Arctium lappa *L. roots on sexual behavior in normal male rats.

**Methods:**

Seventy-five albino male rats were randomly divided into five groups of 15 rats each. Rats in group 1 (control) were administered 10 mL⁄kg body weight distilled water (vehicle), group 2 received 60 mg/kg body weight sildenafil citrate (Viagra), while those in groups 3, 4, and 5 were given 300, 600, and 1,200 mg/kg body weight, respectively, of aqueous extract of *Arctium lappa *L. roots in the same volume. Female albino rats were made receptive by hormonal treatment. Sexual behavior parameters in male rats were monitored on days 3, 7 and 15 by pairing with receptive females (1:3). Male serum testosterone concentrations and potency were also determined.

**Results:**

Oral administration of *Arctium lappa *L. roots extract at 600 and 1,200 mg/kg body weight significantly increased the frequencies of mount, intromission, and ejaculation frequency (*p *< 0.05). The latencies of mount and intromission were significantly reduced and ejaculation latency was prolonged. Administration of the extract also reduced the post-ejaculatory interval. The standard drug (Viagra) was more effective than the extract. The extract significantly increased the frequencies of all components of penile reflexes as well as serum testosterone levels, compared with the distilled water controls.

**Conclusions:**

The results of this study demonstrate that aqueous extract of *Arctium lappa *L. roots enhances sexual behavior in male rats. The aphrodisiac effects of the plant extract may be related to the presence of flavonoids, saponins, lignans and alkaloids, acting via a multitude of central and peripheral mechanisms. These results thus support the traditional use of *Arctium lappa *L. root extract for treating impotence and sterility.

## Background

Sexual relationships are among the most important social and biological relationships in human life. Male sexual dysfunction (MSD) affects not only sexual relationships, but also overall quality of life. MSD includes erectile dysfunction (ED), ejaculation dysfunction, and hypogonadism, and represents a serious public health problem [1]. ED and premature ejaculation (PE) are the two most prevalent male sexual complaints. ED, sometimes called "impotence", is the repeated inability to get or maintain a firm enough erection to allow sexual intercourse [2]. It often has multiple underlying causes, and it has been estimated that around 1 in 10 men will experience recurring impotence problems at some point in their lives [3]. Although ED does not affect life expectancy, it can have a significant negative impact on an individual's well-being and quality of life [4]. PE is the most common sexual dysfunction among young men worldwide, with a prevalence of more than 20% [5,6], and is characterized by a short latency time and a lack of control over ejaculation. In men suffering from PE, not only is the latency to ejaculation typically very short (e.g., 1 or 2 min or less), but the man's perceived control of latency and the timing of ejaculation are low or absent [7]. Experimental studies have demonstrated that the motor control of ejaculation in animals is modulated by serotonin and serotonin receptors [8]. Animal studies have also shown that diminished serotonin neurotransmission and activation of 5-HT_1A _receptors were associated with faster ejaculation, whereas activation of 5-HT_2C _and 5-HT_1B _receptors delayed ejaculation [9,10]. Recent studies have indicated the beneficial effects of some serotonergic antidepressants in delaying ejaculation, and these agents thus comprise the current pharmacological treatment for PE.

MSD is a medical problem that affects both young and old men, and despite advances in modern and orthodox medicines, its effective control by drugs or adjuvant therapies is affected by drug efficacy and safety, as well as cost. Continuing research is therefore needed to develop and investigate safe and effective new drugs for the treatment of MSD. Plants are an important source of medicines and play a key role in the health of the world's population. The use of plant materials to treat sexual disorders has a long history in most countries, and plant materials have proven effective in improving sexual desire and sexual behavior in male animals. For example, *Hypericum perforatum *[11], *Senecio cardiophyllus *[12], *Ginkgo biloba *[13], *Pausinystalia yohimbe *[14], *Fadogia agrestis *[15], *Bryonia laciniosa *[16], *Astercantha longifolia *[17] and *Curculigo orchioides *[18] have all been reported to have sexual-function-enhancing effects in male rats. *Montana tomentosa *is a potent stimulator of sexual behavior, particularly sexual arousal and pro-ejaculatory effects in male rats [19]. *Microdesmis keayana *roots have been reported to enhance sexual behavior in male rats by increasing the production of nitric oxide (NO) [20].

*Arctium lappa *L is a traditional Chinese medicinal and an edible perennial plant of the family Compositae. It has also been used therapeutically in Europe, North America and Asia for hundreds of years. The plant has been cultivated as a vegetable in Japan for many years. *Arctium lappa *L. root is traditionally used in herbal remedies to treat tonsillitis, throat pain, arthritis, rashes, and various skin problems, and as a diuretic, diaphoretic, and blood purifier [21,22]. In Traditional Chinese Medicine, *Arctium lappa *L. root is recommended as an aphrodisiac agent, and used for the treatment of impotence and sterility, while Native Americans included the root in herbal preparations for women in labor [23]. However, these claims are largely based on subjective opinions rather than scientific observations, and the effects of *Arctium lappa *L. root on sexual behavior have not been scientifically validated. This study therefore evaluated the potential aphrodisiac properties of this plant extract by investigating the effects of aqueous extract of *Arctium lappa *L. roots on sexual behavior in male rats.

## Methods

### Chemicals

The drugs used in this study were sildenafil citrate (Viagra) (Pfizer Inc, USA), progesterone (Ningbo, China) and estradiol (Sigma). Olive oil was from Grupo Hojiblanca (Spain). All reagents used were of analytical grade and were supplied by Tianjin Chemical Agent Ltd., China.

### Preparation of aqueous extract of *Arctium lappa *L. roots

Fresh *Arctium lappa *L. roots were washed in tap water and air-dried in the shade. Four kilograms of roots were cut into small pieces and extracted with warm distilled water (DW) (DW:material, 10:1, v/w) twice in an incubator at 80 C for 1.5 h. The hot-water extract was filtered through Whatman No. 1 filter paper (Sanger Biotech, Shanghai, China), and the extraction was repeated. The combined filtrates were concentrated in a vacuum at 60 C, and the resulting filtrates were freeze-dried (Boyikang Refrigerated Vapor Trap, SD-1A-50) and weighed to determine the yield of soluble constituents. The sample was stored at -20 C (yield 12.2% w/w dry weight basis) until use.

### Animals

Adult male Sprague-Dawley rats, approximately 4 months old and weighing 230-260 g, as well as 3-month-old female rats weighing 220-240 g were obtained from the Animal Experimental Center of the College of Medicine, Shandong University. The animals were housed in clean metabolic cages in well-ventilated conditions (temperature 23 ± 2 C; photoperiod: 12 h natural light and 12 h dark; humidity: 45-50%), with free access to standard rat pellets and water.

### Animal groups and extract administration

Seventy-five male rats were randomly divided into five groups of 15 rats each and were orally administered the following: Group 1 (control), 10 mL⁄kg body weight DW; group 2, 60 mg/kg body weight Viagra; groups 3, 4 and 5, 300, 600 and 1,200 mg/kg body weight, respectively, of *Arctium lappa *L. root extract.

Oral administration was carried out using a metal oropharyngeal cannula. Five rats in each group were monitored for sexual behavior after their daily doses on days 3, 7 and 15. The experiments on animals were conducted in accordance with the internationally accepted principles for laboratory animal use and the experimental protocols duly approved by the Institutional Ethical Committee.

### Mating behavior test procedure

Male rats were trained three times with sexually receptive females for sexual experience. Each male rat was allowed a 30-min exposure to a female rat in behavioral estrous for copulatory behavior, as described previously [24,25]. The test was carried out between 19.00 and 23.00 h under dim light. Three receptive female rats were introduced to each male rat in a metabolic cage (48.5 cm × 33.5 cm × 22.5 cm) for 30 min (adaptation period) after drug administration. The female was made receptive by the sequential administration of estradiol benzoate (10 μg⁄100 g body weight) and progesterone (0.5 mg/100 g body weight) by subcutaneous injections at 48 h and 4 h, respectively. Male rats from each group were monitored for sexual behavior for a 40-min observation period after their daily drug doses on days 3, 7, and 15. The following male sexual behavior parameters were recorded or calculated after monitoring for the observation period: mount (MF) and intromission frequencies (IF) (the number of mounts and intromissions from the time of introduction of the female until ejaculation), mount (ML) and intromission latencies (IL) (the time interval between the introduction of the female and the first mount or intromission by the male), ejaculation frequency (EF), ejaculatory latency (EL) (the time interval between the first intromission and ejaculation), and post-ejaculatory interval (PEI) (the time interval between ejaculation and the first intromission of the following series).

### Determination of serum testosterone levels

Serum testosterone levels were determined according to a previously described procedure [26]. Blood was collected about 2 h after administration of the extract, Viagra, or DW. Rats were bled through their cut jugular veins (which were slightly displaced to prevent blood contamination by interstitial fluid) under ether anesthesia, into clean, dry centrifuge tubes. The blood was left for 10 min at room temperature to clot. The tubes were then centrifuged at 3,000 rpm for 10 min using an Anke centrifuge (Model TGL-16 G, Shanghai, China). The serum was aspirated with a Pasteur pipette into a clean, dry, sample bottle and used for testosterone assays within 12 h of preparation. Serum hormone concentrations were determined using the Beckman Coulter Access 2 immunoassay system and a complete set of chemiluminescence reagents (Affiliated Hospital of Shandong University).

### Test for penile reflexes

The effects of the test drug were studied according to the methods described by Tajuddin et al. [26,27]. Male animals were divided into five groups of five animals each and kept singly in separate cages during the experiment. Group 1 represented the control group, which received 10 ml/kg of DW orally. Groups 2-4 received the test drug orally at doses of 300, 600 and 1,200 mg/kg, respectively, daily for 15 days. Group 5 received Viagra orally at 60 mg/kg. The test for penile reflexes was carried out on the 15th day by partially restraining the animal on its back in a glass cylinder. The preputial sheath was pushed behind the glans using the thumb and index finger and held in this manner for 15 min. This stimulation elicited a cluster of genital reflexes, and the frequencies of the following components of penile reflexes were recorded or calculated: erections (E), quick flips (QF), and long flips (LF) were recorded, and total penile reflexes (TPR) were determined as E + QF + LF.

### Statistical analysis

Results were expressed as mean ± S.D.M The significance of difference between the means was determined by one-way analysis of variance (ANOVA) with post hoc tests, followed by analysis with SPSS 13.0 for Windows software. Values were considered significant when *p *< 0.05.

## Results

The observations of sexual behavior are presented in Table 1. Treatment with aqueous extract of *Arctium lappa *L. roots at all three doses influenced the behavior of the treated animals in a dose-dependent manner. Both 600 mg/kg and 1,200 mg/kg body weight significantly affected sexual behavior, compared with the control.

**Table 1:** Effect of aqueous extract of burdock roots on mating behavior in male rats

Sexual- behavior parameters	Days of treatment	**Mean frequency ± S.E.M**.				
		
		Control	Burdock extract 300 mg/kg	Burdock extract 600 mg/kg	Burdock extract 1,200 mg/kg	Viagra (60 mg/kg)
IF	3rd day	10.6 ± 0.8	11.8 ± 1.4	12.0 ± 1.2	13.2 ± 0.9	15.00 ± 1.3*

	7th day	11.4 ± 1.6	13.4 ± 1.5	15.2 ± 1.1	16.2 ± 0.8*	15.8 ± 1.6*

	15th day	10.8 ± 0.9	13.8 ± 1.4	15.0 ± 1.2*	19.4 ± 0.9**	21.6 ± 1.8**

MF	3rd day	13.6 ± 0.9	14.6 ± 2.5	15.4 ± 1.3	17.0 ± 1.4	19.8 ± 1.9*

	7th day	13.4 ± 1.5	17.6 ± 1.5	19.4 ± 2.1*	21.2 ± 2.5*	20.2 ± 2.0*

	15th day	12.8 ± 1.1	16.6 ± 1.5	22.4 ± 1.6**	27.2 ± 2.0**	30.6 ± 2.4**

IL	3rd day	119.2 ± 9.3	117.8 ± 11.4	115.6 ± 8.7	105.0 ± 10.4	89.4 ± 5.9*

	7th day	117.6 ± 7.5	104.0 ± 9.0	89.2 ± 5.1**	78.0 ± 4.5**	64.6 ± 7.1**

	15th day	108.6 ± 11.9	89.8 ± 8.0	78.4 ± 3.7**	59.0 ± 5.0**	55.4 ± 4.6**

ML	3rd day	107.0 ± 5.5	96.4 ± 6.4	93.6 ± 5.2	90.2 ± 7.4	72.0 ± 5.7**

	7th day	100.2 ± 4.9	84.2 ± 3.9	74.2 ± 9.1**	60.4 ± 4.3**	58.6 ± 4.2**

	15th day	94.0 ± 9.6	77.6 ± 8.6	63.6 ± 2.7**	48.2 ± 3.1**	44.4 ± 2.8**

EF	3rd day	1.8 ± 0.4	2.2 ± 0.4	2.4 ± 0.6	2.8 ± 0.4	2.6 ± 0.5

	7th day	2.2 ± 0.3	2.8 ± 0.6	3.0 ± 0.5	3.2 ± 0.6	3.4 ± 0.6

	15th day	2.0 ± 0.3	3.0 ± 0.3	3.4 ± 0.5*	3.6 ± 0.6*	3.8 ± 0.4**

EL	3rd day	213.1 ± 15.1	215.9 ± 16.8	221.6 ± 25.0	231.2 ± 19.4	250.3 ± 13.8

	7th day	220.3 ± 13.3	251.2 ± 15.1	262.3 ± 23.9	281.3 ± 20.5*	284.8 ± 24.7*

	15th day	211.1 ± 13.4	260.3 ± 19.8	294.3 ± 29.7**	329.2 ± 12.4**	335.8 ± 17.7**

PEI	3rd day	414.8 ± 26.2	399.4 ± 28.1	416.5 ± 20.5	410.5 ± 30.3	353.8 ± 16.7

	7th day	439.6 ± 23.5	386.6 ± 32.3	347.9 ± 26.6*	327.4 ± 23.4**	332.5 ± 21.1**

	15th day	426.6 ± 39. 3	345.9 ± 28.5*	297.8 ± 17.4**	268.3 ± 21.9**	258.5 ± 19.4**

ML, mounting latency; IL, intromission latency; EL, ejaculation latency; MF, mounting frequency; IF, intromission frequency; EF, ejaculation frequency; PEI, post-ejaculatory interval. S.E.M.: mean standard error. n = 6 (number of animals in each group), significant difference from control, significance level: *p < 0.05, **p < 0.01

Aqueous extract of *Arctium lappa *L. roots at all three doses had no significant effect on MF or IF on day 3 (*p *> 0.05), but both MF and IF were significantly increased in these groups on days 7 and 15, compared with the distilled-water control (*p *< 0.05). Aqueous extract of *Arctium lappa *L. roots at all the tested doses had no significant effect on EF in male rats on days 3 and 7 (*p *> 0.05). However, EF was increased at day 15 in animals treated with 600 and 1,200 mg/kg body weight extracts (*p *< 0.05). Administration of either 600 or 1,200 mg/kg body weight extract for 7 and 15 days significantly decreased both ML and IL, compared with the distilled-water control (*p *< 0.05). The extract produced contrasting effects on EL and PEI in the male rats. EL increased following a single dose of 1,200 mg/kg body weight, while PEI was significantly decreased at this dose on day 7, compared with the distilled-water control (both *p *< 0.05). Continued administration of the extract at all doses for 15 days decreased PEI in a dose-related manner, whereas EL was increased in a dose-related manner (*p *< 0.05). Throughout the duration of the experiment, precoital sexual behavior (chasing, nosing, anogenital sniffing, genital grooming and attempted clasping and mounting) was notable in the highest-extract-dose group (1,200 mg/kg body weight), though the standard drug (Viagra) was more effective than the extract. Treatment with aqueous extract of *Arctium lappa *L. roots at all three doses remarkably delayed EL without exerting any negative effects on other sexual behavior parameters and with no locomotor alterations throughout the observation period.

Similarly, serum testosterone concentrations were significantly increased by the end of the experimental period in the 600 and 1,200 mg/kg aqueous-extract groups (*p *< 0.05, Table 2). Serum testosterone concentrations were also increased in the Viagra group, but this increase was not significant compared with the control.

**Table 2:** Effect of administration of aqueous extract of burdock roots for 15 days on serum testosterone concentrations

Parameter	Mean frequency ± S.E.M				
	
	Control	Burdock extract 300 mg/kg	Burdock extract 600 mg/kg	Burdock extract 1,200 mg/kg	Viagra (60 mg/kg)
Testosterone	2.3 ± 0.4	3.2 ± 0.5	4.3 ± 0.5*	5.2 ± 0.5**	2.9 ± 0.6

S.E.M.: mean standard error. n = 6 (number of animals in each group), significant difference from control, significance level: *p < 0.05, **p < 0.01

The test for potency showed that TPRs and their components were significantly enhanced by higher doses of *Arctium lappa *L. root extract (600 mg/kg body weight, *p *< 0.05, 1,200 mg/kg body weight, *p *< 0.01, Table 3).

**Table 3:** Effect of aqueous extract of burdock roots on penile reflexes (test for potency)

Parameter	**Mean frequency ± S.E.M**.				
	
	Control	Burdock extract 300 mg/kg	Burdock extract 600 mg/kg	Burdock extract 1,200 mg/kg	Viagra (60 mg/kg)
Erection	6.6 ± 0.9	8.0 ± 1.1	10.2 ± 1.2*	11.2 ± 1.3**	12.4 ± 1.1**

Quick flips	5.2 ± 0.9	6.8 ± 0.9	8.6 ± 1.2*	9.8 ± 1.2**	11.4 ± 1.4**

Long flips	2.3 ± 0.4	3.4 ± 0.8	4.6 ± 0.8*	7.8 ± 1.1**	8.2 ± 0.9**

Total penile reflex	14.1 ± 2.2	18.2 ± 2.8	23.4 ± 3.2*	28.8 ± 3.6**	32.0 ± 3.4**

S.E.M.: mean standard error. n = 6 (number of animals in each group), significant difference from control, significance level: *p < 0.05, **p < 0.01.

## Discussion

This study examined the effect of aqueous extract of *Arctium lappa *L. roots on male sexual competence in rats, with viagra as a positive reference drug. To the best of our knowledge, this is the first study to report the effects of aqueous extract of *Arctium lappa *L. roots in male rodents. *Arctium lappa *L. root extract enhanced the sexual behavior of male rats compared with controls administered DW. These results scientifically support the use of *Arctium lappa *L. roots for enhancing male sexual ability.

The mating behavior test revealed that aqueous extract of *Arctium lappa *L. roots significantly increased MF and IF, compared with the control group, though the effect was less than that of Viagra. Aqueous extract of *Arctium lappa *L. roots also caused significant reductions in ML and IL, compared with control animals, while highly significant decreases in ML and IL were observed in animals treated with Viagra. MF and IF are considered to be indices of libido (sexual desire) and potency, while ML and IL are also indicators of sexual arousal. The significant increases in MF and IF and the decreases in ML and IL indicate that libido and potency were enhanced by *Arctium lappa *L. root extract [28-31]. Furthermore, the prolongation of EL by aqueous extract of *Arctium lappa *L. roots is an indicator of prolonged duration of coitus. PEI is considered to be an index of potency, libido, and the rate of recovery from exhaustion after the first series of mating. The decreased PEI observed with various doses of plant extract may have been the result of enhanced potency and libido, and/or reduced exhaustion in the first series of matings. These observations all further support the role of *Arctium lappa *L. root extract in improving sexual function.

The penile erection index is important for evaluating the effect of drug administration on erectile function [32]. The potency test showed that the extract significantly increased the frequency of all components of the penile reflex compared with the control group, but to a lesser degree than Viagra. This indicates that the aqueous extract of *Arctium lappa *L. roots also increases potency. Treatment with all three doses of aqueous extract of *Arctium lappa *L. roots remarkably delayed EL, with no negative effect on the other parameters of sexual behavior, and with no locomotor alterations throughout the observation period. The delayed EL and increased penile erection in treated male rats indicated the involvement of NO in the intervention [33]. The use of this plant during labor by Native Americans suggests an oxytocic effect of the plant's biologically active components. Oxytocin is known to be a potent facilitator of copulatory behavior in male rats, centered on ejaculatory function [34]. Oxytocin administration has been shown to affect the consummatory components of masculine sexual behavior by lowering the ejaculatory threshold and dramatically reducing EL [35]. However, the physiological mechanism responsible for the involvement of the oxytocinergic system in promotion of sexual potency by the extract cannot be identified based on the results of the current study.

The continued administration of various concentrations of *Arctium lappa *L. roots extract for 15 days increased hormone levels. The mean testosterone level in untreated males was 2.27 ng/mL, and this was significantly increased to 5.18 ng/ml in the group treated with the highest dose of extract (*p *< 0.01). Administration of extract increased testosterone, indicating the involvement of the stimulation of hypothalamic-pituitary-gonadal axis, the increase in testosterone might have been caused by the enhancement in the GnRH-LH signalling [36]. Testosterone is the main male gonadal hormone produced by the interstitial Leydig cells of the testis. In the testes, Luteinizing hormone (LH), a gonadotrophin, which binds to receptors on Leydig cells, stimulating synthesis and secretion of testosterone. Administration of extract increased testosterone could thus be assumed that some phytoconstituent present in the extract may possibly mimic the function of LH to stimulate interstitial cells. In the complex mechanism that regulates copulatory behavior, an increase in testosterone level has been associated with a moderate but significant increase in sexual desire and libido [37-39]. Penile tumescence and rigidity, as well as the accessory muscles that help to provide additional penile rigidity and ejaculation, depend on testosterone for normal sexual activity [40]. Testosterone may also facilitate male sexual behavior by increasing dopamine release in the medial preoptic area and potentiating nitrergic neurotransmission [41,42]. Increased serum testosterone levels after administration of *Arctium lappa *L. roots extract could thus be considered as one of the contributing factors responsible for the overall increased sexual performance in the treated groups, especially for the lengthening of EL and increased copulatory ability in rats. Overall, these results suggest that *Arctium lappa *L. roots extract might represent an interesting alternative to serotonergic antidepressants for the treatment of PE.

Studies in laboratory animals have implicated many components of plant extracts as possible bioactive agents responsible for increasing endogenous testosterone levels and enhancing male sexual behavior. These include steroids and steroidal saponins, which may act as intermediaries in the steroidal pathway of androgen production. Saponins may bind to hormone receptors, resulting in conformational changes that can enhance the physiological functions of the hormone, or can bind to enzymes involved in the synthesis of such hormones, thus enhancing their production [43,44]. In addition, flavonoids have been implicated in altering androgen levels and may also be responsible for enhancing male sexual behavior by enhancing testosterone synthesis or by preventing its metabolic degradation [45,46].

In addition to increasing the biosynthesis and secretion of androgens, many bioactive components of plant extracts also exhibit aphrodisiac activities by acting directly on the central nervous system to modulate the action of neurotransmitters and gonadal tissues in males, or through vasodilation and the generation of NO, which can also change sexual behavior. Alkaloids increase the dilation of blood vessels in the sexual organs [20,47]. Ginseng saponin has been shown to enhance libido and copulatory performance by acting directly on the central nervous system and gonadal tissues [48], and evidence suggests that it can facilitate penile erection by directly inducing the vasodilatation and relaxation of the penile corpus cavernosum via an NO-dependent mechanism [49].

Phytochemical studies indicate that *Arctium lappa *L. root contains sterols, flavonoids, phenols, saponins, lignans (such as arctiin), alkaloids, sugars (polysaccharides), vitamins, tannin, minerals, lactone, polyacetylenes and amino acids [21,22,50,51]. The improvements in sexual function demonstrated in the current study might thus be due to the presence of such compounds in *Arctium lappa *L. root extracts. Further studies are required to identify the active constituent(s) responsible for the sexual function improvement activities and the mechanisms whereby these activities are implemented. The results of the current study suggest that aqueous extract of *Arctium lappa *L. roots may be a promising new agent for the clinical treatment of MSD.

## Conclusions

Overall, this study demonstrated that aqueous extract of *Arctium lappa *L. roots could enhance sexual function and behavior in male rats. These results support the acclaimed use of this plant as an aphrodisiac in Chinese folk medicine. Its aphrodisiac effect may be due to the presence of flavonoids, saponins, lignans and alkaloids acting through a multitude of central and peripheral pathways.

## Competing interests

The authors declare that they have no competing interests.

## Authors' contributions

JianFeng Cao designed the study. PengYing Zhang, ChengWei Xu, TaoTao Huang and Yun Gui Bai participated in the statistical analysis. KaoShan Chen supervised the design of and coordinated the study. All authors read and approved the final manuscript.

## Pre-publication history

The pre-publication history for this paper can be accessed here:

http://www.biomedcentral.com/1472-6882/12/8/prepub
